# Commentary: Getting into predictive processing's great guessing game: Bootstrap heaven or hell?

**DOI:** 10.3389/fpsyg.2017.01244

**Published:** 2017-07-20

**Authors:** Michał Piekarski

**Affiliations:** Institute of Philosophy, Cardinal Stefan Wyszynski University Warsaw, Poland

**Keywords:** content, representation, predictive processing theory of cognition, prediction, action, decision making

Hutto and Myin (2013) put forward a thesis that the so-called basic minds do not have any content. From their point of view, this means that basic minds are not representational. There is no value that can be ascribed to concepts such as “content” and “representation” when it comes to explaining perception and mental processes. Hutto and Myin believe that content is something which is superimposed upon mental processes by means of language and culture. They claim that most contemporary positions in the philosophy of mind and cognitive science assume Content Involving Account of Cognition (CIC). Radical Enactive, Embodied Account of Cognition (REC) rejects the belief in the constitutive role of content in cognition. The radicalism of their approach stems from the failure to solve the Hard Problem of Content (HPC), which undermines the possibility of offering a naturalistic justification of the relation between contentful properties and physical properties (Hutto and Myin, 2013, p. 69).

## Contentful predictions, and contentless expectations

Hutto (2017) claims that the model of cognition developed on the basis of a neurological conception of predictive processing is not HPC-proof either. Representational explanations postulated by the predictive processing do not provide a satisfactory answer to the question about conditions for the possibility of content. Clark, whose position is the main focus of Hutto's criticism, suggests that we should differentiate between two kinds of predictions: “the kind of conscious guessing (…) and the kind of automatically deployed, deeply probabilistic, non-conscious guessing that occurs as part of the complex neural processing routines that underpin and unify perception and action” (Clark, 2016, p. 2). Hutto's proposal is to treat this differentiation as separating contentful predictions made by people and contentless, embodied expectations made by the brain.

I intend to demonstrate in this commentary that (1) it is not possible to think about predictions without referring to the concept of representation (see Gładziejewski, 2016); and (2) Hutto's criticism is based on a wrong interpretation of the two types of prediction identified by Clark. If we accept the existence of a multi-level generative model postulated by predictionists, the division into personal and sub-personal predictions will not be tenable and will not have any explanatory value. I cannot justify, as Hutto is doing, that contentful predictions are derived from contentless expectations. To this end, I will refer to the analyses of cortical mechanisms of action selection made by Cisek (Cisek, 2005, 2007; Cisek and Pastor-Bernier, 2014).

## Decision-making in the ACH model

Cisek proposes to explain perception based decision-making processes on the basis of the ACH model (*Affordance Competition Hypothesis*). According to this model, decisions emerge out of a distributed and probabilistic competence which occurs between multiple representations of possible actions and sensorimotor informations. These decisions are not determined by any specific process instantiated in the brain but by the area of the brain which first commits to a specific action in such a way that it influences other areas. This results in the so-called *distributed consensus*. The right decision is selected through interactions of specific actions undertaken by relevant areas in the brain (starting with rule-based inputs originating in prefrontal regions through reward predictions made by basal ganglia and a range of further biasing variables from sub-cortical regions responsible for the correct functioning of sensory modalities and motor skills). Cisek and Pastor-Bernier stress that neuronal representations serve as indicators for potential actions adapted to the agent's environment (Cisek and Pastor-Bernier, 2014, p. 4. see also: Clark, 2016, p. 177–181).

## Representations and predictions

Burr (2017) believes that the ACH model dovetails with and is complemented by the predictive processing framework. Under the prediction processing, neuronal representations of actions are qualified as predictions which may change depending on the unstable and uncertain environment. Such representations are understood as particular patterns of neural activation. Representation in the ACH model, however, have satisfaction conditions, that is they are directly involved in the process of minimizing the prediction error (active reasoning) (Gallese and Metzinger, 2003). This does not mean, however, that the conception stands the test of Hutto and Myin's objection. Hutto is right—having conditions of satisfaction is not sufficient to bar the HPC. Importantly, the representations postulated by the ACH model can be specifically applied to explain perceptual processes. They are rooted in specific facts related to the embodiment of a given cognitive system. We should therefore conclude that the decision-making process must be understood dynamically: the process of action selection in ACH unfolds fluently at different levels of the hierarchy which track environmental and bodily regularities at different time scales. Thus, a specific decision is made on the basis of: (1) information coming from the sensory signal, (2) representations of actions which are predictions, and (3) an uncertain and changeable environment. I need to agree with Clark who claims: “the picture that here emerges is one of neural encodings that are *fundamentally* in the business of action control. Such encodings represent how the world is in ways that are entwined, at multiple levels, with information about how to act upon the world” (Clark, 2016, p. 181).

## Concluding remarks

The existence of a multi-level generative model postulated by predictionists may guarantee that predictions made at lower levels of the model depend on the content of predictions made at higher levels. Such an approach obliterates Hutto's division into contentful predictions present at the personal level and contentless expectations made by the brain. Given this perspective, it seems that there is no explanatory value in the distinction between the personal and sub-personal predictions, which may suggest that its function is purely descriptive. The above remarks provide a good point of departure for demonstrating that the predictive processing framework is HPC-resistant. Miłkowski's analysis of mental representations (Miłkowski, 2015a,b, 2016) also strengthens the claim about the role of satisfaction conditions in the predictive processing. He shows that content is causally relevant for the functioning of the cognitive anticipatory representational mechanisms because it allows for correction of erroneous predictions.

## Author contributions

MP reviewed the literature and developed the theoretical stance and wrote the manuscript and prepared to publication.

### Conflict of interest statement

The author declares that the research was conducted in the absence of any commercial or financial relationships that could be construed as a potential conflict of interest. The reviewer KD and handling Editor declared their shared affiliation, and the handling Editor states that the process nevertheless met the standards of a fair and objective review.
